# Determinants of Willingness to Receive Health Information From Neighborhood Food and Beauty Establishments: Cross-Sectional Study

**DOI:** 10.2196/86435

**Published:** 2026-05-14

**Authors:** Seema Aithal, Huiling Guo, Zoe Lara-Jane Hildon, May O Lwin, Angela Chow

**Affiliations:** 1 Department of Epidemiology and Preventive Medicine, Tan Tock Seng Hospital, 11 Jalan Tan Tock Seng, Singapore, 308433, Singapore, 65 63577477; 2 Saw Swee Hock School of Public Health, National University of Singapore, Singapore, Singapore; 3 Wee Kim Wee School of Communication and Information, Nanyang Technological University, Singapore, Singapore

**Keywords:** health promotion, health information, trust, willingness, non–health care services

## Abstract

**Background:**

Although health care providers are the most trusted sources of health information, service establishments within communities represent important, yet underused, sources of health information. Specifically, food and beauty establishments can act as alternative settings for health communication, facilitating broader engagement with the general population.

**Objective:**

This study examined factors associated with willingness to receive health information from these non–health care service establishments among community-dwelling adults in Singapore.

**Methods:**

A cross-sectional survey was conducted among residents in 2 neighborhoods in central Singapore between November 2024 and April 2025. Data on sociodemographic characteristics, trust in information from health care and non–health care services, and willingness to receive health information were collected anonymously. The primary outcome was willingness to receive health information from non–health care services (yes or no), assessed among respondents with no prior exposure to health information from such services. Multivariable logistic regression was used to identify factors independently associated with willingness to receive health information from non–health care services.

**Results:**

Among the 403 respondents, most were aged ≥50 years (n=223, 55.3%), female (n=219, 54.3%), Chinese (n=350, 86.9%), and highly educated (n=302, 74.9%). Of the 339 respondents without prior exposure to health information from non–health care services, approximately one-third (n=106, 31.3%) reported that they were willing to receive health information in the future. In adjusted analysis, greater trust in health information (adjusted odds ratio [AOR] 3.71, 95% CI 1.50-9.19) and high health information orientation (AOR 1.89, 95% CI 1.11-3.21) were associated with increased willingness to receive health information from non–health care services. Trust in health information was positively associated with willingness among those aged 21 to 34 years (AOR 4.96, 95% CI 1.35-18.30), those aged 35 to 49 years (AOR 8.02, 95% CI 2.62-24.59), and male respondents (AOR 6.22, 95% CI 2.79-13.89) to receive health information from these sources, but not among those aged ≥50 years (AOR 1.92, 95% CI 0.92-4.02) or female respondents (AOR 1.85, 95% CI 0.87-3.96).

**Conclusions:**

Nearly one-third of community-dwelling adults expressed willingness to receive health information from non–health care (food and beauty) services, highlighting the potential for leveraging these establishments as alternative health communication channels. Willingness was positively associated with higher health information orientation and greater trust. Additionally, trust in non–health care (food and beauty) services was associated with higher odds of willingness to receive health information among those aged 21 to 49 years and male respondents. This suggests the need for tailored trust-building strategies to strengthen engagement through such alternative channels.

## Introduction

According to the World Health Organization, health promotion is defined as the process of enabling people to increase control over and improve their health by fostering activities that enhance the biological, social, psychological, environmental, and economic conditions necessary for achieving optimal health [1]. Health education is a central component of health promotion and empowers populations to adopt healthy behaviors [2]. Importantly, Sørensen et al [3] highlight that access to health information represents the essential first step before individuals can understand, evaluate, and apply such information to adopt desirable health behaviors.

Although health care providers represent the primary trusted information sources [4,5], equitable access to health information continues to pose significant challenges, especially for specific subpopulations and individuals with unique biopsychosocial needs [6]. Individuals who turn first to their health care providers for health information represent a unique population and mainly comprise older adults (aged >65 years), those in poor health, those with lower education, those having health insurance, and non–internet users [7]. This underscores the need to explore nontraditional approaches for disseminating health information across the wider community, including service establishments. Such approaches may be especially relevant for younger adults, who typically have fewer health care encounters owing to lower chronic disease burden, thereby emphasizing the importance of community-based social networks and touchpoints for engaging this demographic [8].

The African American barbershop health initiative in the United States illustrates the successful transformation of non–health care community spaces into health promotion venues by tapping into existing social networks and trusted relationships with barbers serving as community health advocates [9,10]. The initiative’s success stemmed from recognizing barbershops as natural gathering spaces where health messages could be effectively conveyed in familiar, comfortable settings. This approach has been efficacious across multiple health areas, including outreach for infectious diseases (HIV and COVID-19), chronic disease management (diabetes and hypertension), cancer screening, and mental health promotion [11-18]. Furthermore, this community-based health promotion approach has proven remarkable adaptability across varied cultural and health care contexts internationally [19-25], attesting to the viability of engaging non–health care services for health promotion and education outreach.

Food and beauty establishments can serve as alternative venues for disseminating health information and expanding outreach to the general population. Beauty establishments, including barbershops and beauty salons, have been heavily studied, but little is known about the utility of other non–health care establishments in the community for disseminating health information at scale. In Singapore, where an estimated 60% of residents dine out regularly [26], hawker centers, food courts, and coffee shops often serve as “community dining rooms” that cater to all ages, classes, and schedules [27]. Programs promoting healthier diets and hawker center etiquette, mainly targeting patrons of these food establishments, have been successfully implemented [28,29]. Moreover, hawkers often recognize their regular patrons and keep up with their patrons’ lives [30]. The potential of neighborhood establishments with high patronage, specifically food and beauty establishments, to serve as conduits for public health messaging warrants consideration. Such venues may be especially valuable for reaching out to younger demographics with infrequent health care contact while tapping into existing social connections formed in these community spaces.

Trust constitutes an important prerequisite for individuals’ willingness to receive health information from non–health care sources. Prior research has shown that trust reduces perceived risk [31], implying that individuals with greater trust in the information provider are less likely to view the information as inappropriate or potentially harmful compared with those who have lower trust. Moreover, trust has also been shown to positively influence participant compliance and cooperation [32]. For example, a Dutch study [33] investigating the association between trust in health insurers and willingness to receive health care advice from these insurers found that higher levels of trust were significantly associated with increased willingness to receive health care advice. Against this backdrop, this study aims to explore the willingness of community-dwelling adults in Singapore to receive health information from non–health care services, specifically food and beauty establishments located within residential neighborhoods, and identify the factors that influence it.

## Methods

### Study Design and Setting

A cross-sectional survey was conducted in 2 neighborhoods located in central Singapore between November 2024 and April 2025. The sampling frame comprised all residential units located within the 2 selected neighborhoods, identified from the Housing and Development Board residential database and verified against existing physical mailboxes. After excluding units located above commercial premises, a survey package consisting of a study information sheet (appended with a URL or QR code for access to the online survey) and a step-by-step instructional infographic guide was delivered to all eligible residential units by postal mail. Each household received a participant information sheet containing a unique identifier to detect duplicate submissions and facilitate participant reimbursement.

The online self-administered survey included predominantly close-ended questions with a predefined set of responses. Participants were required to complete all questions in the survey to ensure valid submission and minimize item nonresponse. Only 1 resident per household, aged ≥21 years, was eligible to participate in the study. Initial recruitment involved invitations to 9913 households within the study area. To boost participation, reminder letters were sent to 500 randomly selected households from the list of remaining addresses that had not yet responded to the initial survey invitation. Subsequently, research assistants conducted door-to-door follow-up at these 500 household addresses to achieve the target minimum sample size of 385 participants. Assistance was provided to older participants who expressed interest but faced difficulties with self-completion, through interviewer administration.

### Ethical Considerations

The study was approved by the National Healthcare Group Domain Specific Review Board of Singapore (2023/00118). Informed consent was waived, and participants provided implied consent by voluntarily completing and submitting the online survey. Participants who completed the survey received S$5 (US $3.93) as reimbursement for their time. No personal identifiers were collected in the survey, and deidentified data were used for analysis.

### Survey Instrument

The survey questionnaire collected comprehensive sociodemographic information, including age, sex, ethnicity, marital status, highest educational qualification, employment status, and duration of residency in the neighborhood (<10 years or ≥10 years). Questions on self-reported health status (very good and good vs fair, poor, and very poor), health information orientation, and health literacy were also included. Health information orientation [34], which describes an individual’s inherent interest in actively seeking relevant health information to make informed decisions about their health, was assessed using 8 items measured on a 5-point Likert scale (1=strongly disagree to 5=strongly agree), with scores ≥32 (≥80% of the maximum score of 40) indicating a high level of health orientation. Health literacy [35] was evaluated using a 16-item European Health Literacy Survey Questionnaire Short form, where responses were dichotomized (very difficult or difficult=0 and easy or very easy=1), and a total score of ≥13 was classified as an adequate level of health literacy.

The survey also incorporated questions on whether participants passively received health information and actively sought health information from a range of sources, including health care professionals, staff from community care centers, social contacts (family members, friends, and neighbors), social influencers or bloggers, and staff from food and beauty establishments. Data were also collected on residents’ passive receipt of information across 5 different types of health topics: health screening, lifestyle behaviors, chronic disease management, vaccination, and antibiotic use guidance. Trust was operationalized as overall trust in health information received from various sources, including health care clinics and non–health care (food and beauty) establishments. It was assessed using a 5-point Likert scale (1=never, 2=somewhat, 3=moderately, 4=a lot, and 5=completely), and participants who selected “moderately,” “a lot,” or “completely” were deemed to have trust in health information from health care and non–health care services. The questionnaire was developed in English and translated into 2 other local languages: Mandarin and Malay (the survey questionnaire is available in Multimedia Appendix 1).

### Data Analysis

The primary outcome was future willingness to receive health information from non–health care services (yes or no), evaluated exclusively among those who had never previously received health information from such services. In accordance with the study hypothesis, non–health care services were operationally defined as food and beauty establishments. Participants who reported prior receipt were excluded to avoid potential confounding, as previous exposure could reasonably influence both their trust in health information from these services and their willingness to receive health information in the future.

Descriptive analysis involved categorical variables expressed as proportions and continuous variables as means with SDs. Chi-square test was used to compare differences between the 2 outcome groups. First, we conducted univariate analysis to inform variable selection for subsequent multivariable analysis. Covariates were identified a priori through a review of the literature and selected based on their relevance as determined by the research team. The initial multivariable logistic regression model adjusted for core sociodemographic variables (age group, gender, and ethnic group), along with variables that were significant or showed marginal significance (*P*<.20) in univariate analysis. Next, potential interactions between trust and other covariates were systematically explored based on theoretical considerations. Significant interaction terms were sequentially added in the subsequent models. Multicollinearity was assessed using the variance inflation factor. The final regression model was selected based on goodness-of-fit criteria, including Akaike information criteria and log likelihood ratio tests (Table S1 in Multimedia Appendix 1). Additional analyses explored effect modification by age and gender. Statistical significance was defined as *P* <.05, with all analyses conducted using Stata (version 18.0; StataCorp LLC).

## Results

In total, 406 survey responses were collected from 9913 households, giving an overall response rate of 4.1%. After data cleaning, 403 responses were included in the final analysis sample, with 3 (0.01%) excluded due to duplicate responses from the same households.

### Cohort Characteristics

Among the 403 respondents, 54.3% (n=219) were female, with a mean age of 50.9 (SD 15) years. According to the 2025 population census for the 2 study neighborhoods, the demographic profile of respondents was similar to the underlying population in terms of sex (male participants: n=20,310, 46.7% vs female participants: n=23,200, 53.3%; *P*=.68) and age distribution (21‐34 years: n=7910, 18.2%; 35‐49 years: n=10,380, 23.9%; ≥50 years: n=25,220, 58%; *P*=.06). Most respondents were Chinese (n=350, 86.9%), highly educated (n=302, 74.9%), and currently employed (n=270, 67%). Approximately two-thirds (n=262, 65%) were long-term residents with ≥10 in their neighborhoods. Roughly two-thirds of respondents (n=258, 64%) showed high health information orientation and 71.5% (n=288) displayed an adequate level of health literacy. Self-reported health status was fair, poor, or very poor among 41.4% (n=167) of respondents (Table 1). The proportion reporting fair, poor, or very poor health was 35.9% (n=23) among younger adults, 39.7% (n=46) among those aged 35 to 49 years, and 44% (n=98) among those aged ≥50 years.

**Table 1.** Sociodemographic characteristics of respondents from 2 neighborhoods in central Singapore (N=403).

Variables	Respondents
Age (years), mean (SD)	50.9 (15.0)
Age group (years), n (%)	
21‐34	64 (15.9)
35‐49	116 (28.8)
≥50	223 (55.3)
Sex, n (%)	
Male	184 (45.7)
Female	219 (54.3)
Ethnic group, n (%)	
Chinese	350 (86.8)
Non-Chinese	53 (13.2)
Education, n (%)	
Lower education (postsecondary education and below)	101 (25.1)
Higher education (diploma and above)	302 (74.9)
Marital status, n (%)	
Never married, widowed, divorced, or separated	265 (65.8)
Married	138 (34.2)
Employment status, n (%)	
Not currently working	133 (33.0)
Currently working	270 (67.0)
Duration of residency in the neighborhood (years)**,** n (%)	
<10	141 (35.0)
≥10	262 (65.0)
Self-reported health status, n (%)	
Fair, poor, and very poor	167 (41.4)
Good and very good	236 (58.6)
Health information orientation^a^, n (%)	
Low level	145 (36.0)
High level	258 (64.0)
Health literacy^b^, n (%)	
Inadequate	115 (28.5)
Adequate	288 (71.5)
Trust in health information from health care sources, n (%)	
No	18 (4.5)
Yes	385 (95.5)
Trust in health information from non–health care (food and beauty) services, n (%)	
No	298 (74.0)
Yes	105 (26.0)
Ever received health information from health care sources, n (%)	
No	72 (17.9)
Yes	331 (82.1)
Ever received health information from non–health care (food and beauty) services, n (%)	
No	339 (84.1)
Yes	64 (15.9)

a High health information orientation was defined as a score of ≥80% of the total score of 40 (cutoff ≥32).

b Adequate health literacy was defined using the European Health Literacy Survey Questionnaire–Short form (HLS-EU-Q16) standard cutoff score of ≥13.

### Receipt of and Trust in Health Information

Table 2 shows the proportion of respondents who received information both actively and passively stratified by type of information source. Approximately four-fifths of respondents (n=331, 82.1%) had passively received health information from health care sources, but only 11.9% (n=48) and 11.7% (n=47) of respondents indicated prior receipt of health information from food and beauty establishments, respectively. Overall, 16% (64/403) of respondents reported previous receipt of health information from non–health care (food and beauty) services. Information on healthy lifestyle behaviors was the most commonly received health topic from non–health care (food and beauty) services (n=33, 8.2% to n=35, 8.7%) among the different health topics examined. Nearly three-quarters of respondents (n=313, 77.7%) reported having actively sought information from health care clinics for health-related matters, but a lower proportion of respondents had obtained health information from non–health care services (n=14, 3.5% from food establishments and n=16, 4% from beauty establishments). Overall, 95.5% (n=385) of the respondents expressed trust in health information from health care sources as compared to 23.1% (n=93) for food establishments and 20.6% (n=83) for beauty establishments (Table 2).

**Table 2.** Proportion of respondents who passively received, actively sought, and trusted health information, by source type (N=403).

Type of information source	Health care establishments, n (%)	Food establishments, n (%)	Beauty establishments, n (%)	Community care centers, n (%)	Social contacts, n (%)	Social influencers or bloggers, n (%)
Proportion who passively received information on health-related matters	331 (82.1)	48 (11.9)	47 (11.7)	123 (30.5)	189 (46.9)	38 (9.4)
Proportion who passively received information on						
Healthy lifestyle behaviors	260 (64.5)	35 (8.7)	33 (8.2)	81 (20.1)	162 (40.2)	32 (7.9)
Health screening	272 (67.5)	25 (6.2)	23 (5.7)	90 (22.3)	139 (34.5)	17 (4.2)
Appropriate use of antibiotics	260 (64.5)	23 (5.7)	18 (4.5)	70 (17.4)	102 (25.3)	16 (4.0)
Vaccination	253 (62.8)	27 (6.7)	22 (5.5)	80 (19.9)	120 (29.8)	16 (4.0)
Management of chronic diseases	244 (60.5)	26 (6.4)	19 (4.7)	82 (20.3)	128 (31.8)	14 (3.5)
Proportion who actively sought information on health-related matters	313 (77.7)	14 (3.5)	16 (4.0)	49 (12.2)	50 (12.4)	5 (1.2)
Proportion who trusted information on health-related matters	385 (95.5)	93 (23.1)	83 (20.6)	278 (69.0)	179 (44.4)	51 (12.6)

### Willingness to Receive Health Information From Non–Health Care Services

Among the 339 respondents who had not previously received any health information from non–health care (food and beauty) services, nearly one-third (n=106, 31.3%) indicated their willingness to receive such information in the future. Respondents expressed the highest preference for receiving information on chronic disease management from food (99/377, 26.3%) and beauty (91/384, 23.7%) establishments, compared with other health topics they were willing to receive in the future (Table S2 in Multimedia Appendix 1).

A higher proportion of respondents who were willing to receive health information from non–health care (food and beauty) services exhibited high levels of health information orientation (74/106, 69.8% vs 133/233, 57.1%; *P*=.03) and trusted the health information from non–health care services (40/106, 37.7% vs 36/233, 15.5%; *P*<.001) compared to those who were unwilling (Table 3). No significant differences were observed between willing and unwilling respondents with regard to age, sex, ethnicity, or education levels.

**Table 3.** Logistic regression analysis of factors associated with willingness to receive health information from non–health care (food and beauty) services among respondents across 2 neighborhoods in central Singapore (N=339).

Variables	Willingness to receive health information from non–health care services		Univariate model		Multivariable model	
	Yes (n=106)	No (n=233)	Crude OR^a^ (95% CI)	*P* value^b^	Adjusted OR (95% CI)	*P* value^b^
Age (years), mean (SD)	48.2 (14.6)	51.6 (14.9)	—^c^	—	—	—
Age group (years), n (%)						
21‐34	22 (20.8)	32 (13.7)	1.75 (0.93-3.28)	.08	1.30 (0.57-2.99)	.53
35‐49	32 (30.2)	69 (29.6)	1.18 (0.69-1.99)	.54	1.03 (0.53‐2.03)	.92
≥50	52 (49.0)	132 (56.7)	Reference		Reference	
Sex, n (%)						
Male	51 (48.1)	114 (48.9)	Reference		Reference	
Female	55 (51.9)	119 (51.1)	1.03 (0.65‐1.64)	.89	1.41 (0.78‐2.53)	.25
Ethnic group, n (%)						
Chinese	91 (85.8)	209 (89.7)	Reference		Reference	
Non-Chinese	15 (14.2)	24 (10.3)	1.44 (0.72‐2.86)	.30	1.45 (0.68‐3.07)	.33
Education, n (%)						
Lower education (postsecondary education and below)	31 (29.2)	54 (23.2)	Reference		—	
Higher education (diploma and above)	75 (70.8)	179 (76.8)	0.73 (0.44‐1.22)	.23	—	—
Marital status, n (%)						
Never married, widowed, divorced, or separated	66 (62.3)	157 (67.4)	Reference		—	
Married	40 (37.7)	76 (32.6)	1.25 (0.78‐2.02)	.36	—	—
Employment status, n (%)						
Not currently working	35 (33.0)	80 (34.3)	Reference		—	
Currently working	71 (67.0)	153 (65.7)	1.06 (0.65‐1.73)	.81	—	—
Duration of residency in the neighborhood (years), n (%)						
<10	33 (31.1)	90 (38.6)	Reference		Reference	
≥10	73 (68.9)	143 (61.4)	1.39 (0.85‐2.27)	.18	1.72 (0.98‐3.03)	.06
Self-reported health status, n (%)						
Fair, poor, or very poor	44 (41.5)	104 (44.6)	Reference		—	
Good or very good	62 (58.5)	129 (55.4)	1.14 (0.71‐1.81)	.59	—	—
Trust in health information from non–health care (food and beauty) services, n (%)						
No	66 (62.3)	197 (84.6)	Reference		Reference	
Yes	40 (37.7)	36 (15.4)	3.32 (1.95‐5.63)	*<*.*001*	3.71 (1.50‐9.19)	*.005*
Health information orientation^d^, n (%)						
Low level	32 (30.2)	100 (42.9)	Reference		Reference	
High level	74 (69.8)	133 (57.1)	1.74 (1.07‐2.84)	*.03*	1.89 (1.11‐3.21)	*.02*
Health literacy^e^, n (%)						
Inadequate	26 (24.5)	69 (29.6)	Reference		—	
Adequate	80 (75.5)	164 (70.4)	1.29 (0.77‐2.19)	.33	—	—
Interaction between trust in health information from non–health care (food and beauty) services and respondents aged 21 to 34 years	—	—	—	—	3.43 (0.72‐16.37)	.12
Interaction between trust in health information from non–health care (food and beauty) services and respondents aged 35 to 49 years	—	—	—	—	5.42 (1.31‐22.40)	*.02*
Interaction between trust in health information from non–health care (food and beauty) services and female respondents	—	—	—	—	0.23 (0.07‐0.74)	*.01*

a OR: odds ratio.

b Italicized values indicate statistical significance of *P*<.05.

c Not applicable.

d High health information orientation was defined as a score ≥80% of the total score of 40 (cutoff ≥32).

e Adequate health literacy was defined using the European Health Literacy Survey Questionnaire - Short form (HLS-EU-Q16) with a cutoff score of ≥13.

### Determinants of Willingness to Receive Health Information From Non–Health Care Sources

Health information orientation (*P*=.03) and trust (*P*<.001) were the significant predictors of willingness to receive health information in the future from non–health care services such as food and beauty establishments. Compared to those with low health information orientation, respondents with high health information orientation were associated with greater willingness (adjusted odds ratio [AOR] 1.89, 95% CI 1.11‐3.21) to receive health information from non–health care (food and beauty) services. Respondents who trusted health information from non–health care (food and beauty) services were associated with higher odds of willingness to receive health information from such services in the future (AOR 3.71, 95% CI 1.50‐9.19) compared to those who did not trust health information from non–health care (food and beauty) services (Table 3).

Age and sex demonstrated significant interaction effects with trust in health information from non–health care (food and beauty) services. Among respondents aged 21 to 34 years and 35 to 49 years, trust in health information from non–health care (food and beauty) services was associated with nearly 5-fold (AOR 4.96, 95% CI 1.35-18.30) and 8-fold (AOR 8.02, 95% CI 2.62-24.59) higher odds of willingness to receive health information, respectively, after adjusting for sex, ethnicity, long-term residency, and health information orientation. However, this association was not significant among respondents aged ≥50 years (AOR 1.92, 95% CI 0.92-4.02; Table 4). Similarly, sex-stratified analysis showed that trust in health information from non–health care (food and beauty) services was associated with 6-fold higher odds of willingness to receive health information among male respondents (AOR 6.22, 95% CI 2.79-13.89), but not among female respondents (AOR 1.85, 95% CI 0.87-3.96), after adjusting for age, ethnicity, long-term residency, and health information orientation (Table 5).

**Table 4.** Association of willingness to receive health information and trust in health information from non–health care (food and beauty) services, according to age group (N=339).

Willingness to receive health information from non–health care (food and beauty) services	Respondents aged 21 to 34 years		Respondents aged 35 to 49 years		Respondents aged ≥50 years	
	Odds ratio (95% CI)	*P* value^a^^,^^b^	Odds ratio (95% CI)	*P* value^a^^,^^b^	Odds ratio (95% CI)	*P* value^a^^,^^b^
Unadjusted analysis						
Trust in health information from non-healthcare services (yes)	4.50 (1.26-16.04)	*.02*	7.18 (2.40‐21.47)	<.*001*	2.08 (1.01‐4.29)	*.05*
Adjusted analysis**^c^**						
Trust in health information from non-healthcare services (yes)	4.96 (1.35-18.30)	*.02*	8.02 (2.62‐24.59)	<*.001*	1.92 (0.92‐4.02)	.08

a Multiplicative scale.

b Italicized values indicate statistical significance of *P*<.05.

c Adjusted for gender, ethnicity, duration of residency in the neighborhood, and health information orientation.

**Table 5.** Association of willingness to receive health information and trust in health information from non–health care (food and beauty) services, according to sex (N=339).

Willingness to receive health information from non–health care services	Male respondents		Female respondents	
	Odds ratio (95% CI)	*P* value^a^^,^^b^	Odds ratio (95% CI)	*P* value^a^^,^
Unadjusted analysis				
Trust in health information from non-healthcare services (yes)	6.35 (2.90-13.91)	*<.001*	1.81 (0.86-3.80)	.12
Adjusted analysis^c^				
Trust in health information from non-healthcare services (yes)	6.22 (2.79-13.89)	*<.001*	1.85 (0.87-3.96)	.11

a Multiplicative scale.

b Italicized values indicate statistical significance of *P*<.05.

c Adjusted for age, ethnicity, duration of residency in the neighborhood, and health information orientation.

## Discussion

### Principal Findings

This study set out to examine the potential of non–health care service establishments within communities that function as routine, everyday touchpoints for residents and offer place-based opportunities for health communications. Our findings offer valuable insights into the willingness of community-dwelling adults to accept neighborhood non–health care (food and beauty) services as potential health information sources. Of interest, 16% (64/403) of the respondents reported having received health information passively from either food or beauty establishments in their neighborhoods. Among these, information on healthy lifestyle behaviors constituted the largest proportion , compared with health screening, appropriate antibiotic use, vaccination, and chronic disease management. However, nearly a quarter of respondents (83/385, 21.6% to 99/377, 26.3%) with no prior exposure to health information from such sources indicated their willingness to receive information on various health topics in the future. Thus, to harness this potential resource more effectively, it is imperative to first explore the factors that influence the involvement of food and beauty establishments in disseminating health information.

Of the 339 (84.1%) respondents who had never received health information from food and beauty establishments, nearly one-third (n=106, 31.3%) expressed willingness to receive such information from these sources in the future. This finding highlights a meaningful opportunity and suggests that food and beauty establishments may offer some untapped potential as alternative settings for health promotion. By providing health information to receptive audiences in informal, everyday settings, these establishments could complement the traditional health care communication strategies and help broaden the reach of public health messaging.

The results also revealed that higher health information orientation and greater trust were positively associated with an individual’s willingness to receive health information from non–health care (food and beauty) services. It is possible that individuals with high health information orientation are significantly more health conscious and more likely to seek health information from diverse sources compared to those with low health information orientation, who are less inclined to seek health information [36]. Such individuals often supplement the information provided by health care professionals with additional sources [37]. Furthermore, it is suggested that people who actively seek and value health information tend to be more engaged in their communities and more likely to participate in community health activities [38]. Thus, leveraging these individuals as health ambassadors may help to engage peers, build trust, and boost community participation in preventive health interventions.

In line with previous research, we found that the vast majority (n=385, 95.5%) of respondents trusted health information from health care establishments, whereas only a small proportion (n=83, 20.6% to n=93, 23.1%) trusted health information from food and beauty establishments. Prior literature indicated that expertise, accuracy, and credibility or reliability were the most important qualities for trusting information sources [39]. Trust in health care staff is rooted in their academic qualifications, medical knowledge, adherence to professional standards, and high level of authority [40]. Conversely, trust in non–health care sources such as family and friends relies on repeated interactions built over time through familiar relationships and is valued primarily for personal relevance, reassurance, and emotional support [41,42]. Social connections, based on familiarity, often exert significant influence on individual decision-making processes, especially regarding health care decisions [43]. Fostering trust may be an important component of strategies to enhance willingness, and future studies could investigate how trust operates within familiar social networks and how novel interpersonal sources of health information are used and perceived.

Regarding trust in health information from non–health care services, previous research suggests that disseminated messages should align with, and ideally be endorsed by, trusted health care authorities such as the Health Promotion Board and other relevant institutions [4]. This alignment is especially important in the current digital landscape, which is characterized by widespread misinformation amid vast amounts of online content. Consistent and endorsed health messaging could significantly enhance the perceived credibility of non–health care establishments as emerging health advocates within the community. In addition, leveraging the trust fostered through repeated interactions and social relationships between patrons and establishments [43,44] may further enhance the public’s willingness to receive health information from these establishments. This underscores the potential value of engaging food and beauty establishments with high footfall for community health promotion efforts. Given the observed age and gender differences in trust, future research is warranted to explore the mechanisms that foster trust, particularly among younger and male respondents in relation to food and beauty establishments. Such insights could guide strategies for effectively using these venues as community-based health information sources.

Our study has several strengths. To our knowledge, it is the first study to explore the feasibility of using non–health care community services—specifically food and beauty establishments—as channels for public health messaging in a Southeast Asian context, thereby contributing to the limited research in this area. The findings offer useful insights for developing community-based interventions that leverage these non–health care service providers as alternative health communication channels. In particular, the role of non–health care service providers, especially food establishments, extends beyond local relevance and has broader public health implications, given the increasing prevalence of eating-out cultures in both Western and Southeast Asian societies [45]. As such, the insights generated may be applicable to other urban and sociocultural settings. Finally, the use of validated measurement scales for health information orientation and health literacy strengthens the methodological rigor and enables comparison with existing literature.

However, several limitations warrant consideration. First, the cross-sectional design restricts causal inference, thus limiting findings to associational relationships. The possibility of reverse causation or unmeasured confounding also cannot be ruled out due to the cross-sectional nature of our study. Second, although the survey response rate was low, comparisons with available census data from the 2 study neighborhoods on sex and age suggest that selection bias (if any) is likely to be minimal. Third, although trust is context dependent, we did not collect data on the factors that help to build rapport or trust, such as the nature of the interactions between patrons and establishments, or on the alignment of messages with the preferences of the different target audiences. These factors may influence how trust is formed and maintained in real-world settings. Finally, trust in health information from various sources was measured using a single 5-point Likert scale question, without exploring the different dimensions of trust. Future studies should investigate the complex interplay between cognitive and behavioral components of trust across different health information sources, while also considering diverse health conditions and disease severity in naturalistic settings.

### Conclusions

Our findings highlight the potential for using non–health care services, particularly food and beauty establishments located within residential neighborhoods, as channels for health communication, with nearly 1-in-3 community-dwelling adults expressing willingness to receive health information from these sources. Individuals exhibiting higher health information orientation and greater trust were associated with increased willingness. Fostering trust could further improve their willingness to receive health information disseminated through such establishments. Additionally, trust in non–health care services was associated with greater willingness among adults aged 21 to 49 years and among male respondents, but not among those aged ≥50 years or female respondents. Further research is warranted to explore the age- and gender-specific approaches that strengthen trust and subsequently enhance willingness to receive health information from non–health care service establishments.

## Supplementary material

10.2196/86435 Multimedia Appendix 1 Supplementary tables and survey questionnaire used in the study.

## Abbreviations

AOR: adjusted odds ratio
