# Supplementary material for: Determinants of Willingness to Receive Health Information From Neighborhood Food and Beauty Establishments: Cross-Sectional Study
Source: JMIR Public Health Surveill. 2026 May 14;12:e86435. doi: 10.2196/86435 (PMC13175523; doi:10.2196/86435)
Supplement: Multimedia Appendix 1 [file publichealth-v12-e86435-s001.pdf]

Supplementary Table 1 – Variable selection for final multivariable logistic regression model assessing factors associated with willingness to receive health information from non-healthcare (food & beauty) services among respondents across two neighbourhoods in Central Singapore (N=339).

**Model Number**	**Variables in the model**	**AIC**	**BIC**	**-2LL**	***P*-value+**	**Remarks**
M1	Age group, gender, ethnic group	427.4777	446.6077	417.4778	-	Age, gender and ethnic group added regardless
M2	Age group, gender, ethnic group, **long term residency, health information orientation, trust in health information from non-healthcare (food & beauty) services**	405.7783	436.3863	389.7784	**<0.001**	Added significant variables from univariate analysis (including those with P<0.20).
M3	Age group, gender, ethnic group, long term residency, health information orientation, trust in health information from non-healthcare (food & beauty) services, **gender* trust in health information from non-healthcare (food & beauty) services**	403.0859	437.5199	385.086	**0.030**	
M4**	Age group, gender, ethnic group, long term residency, health information orientation, trust in health information from non-healthcare (food & beauty) services, gender* trust in health information from non-healthcare (food & beauty) services, **age group* trust in health information from non-healthcare (food & beauty) services**	400.4163	442.5023	378.4164	**0.036**	

+ Log likelihood-ratio tests between models M2 and M1, M3 and M2 and M4 and M3 respectively

**Final model

Supplementary Table 2: Willingness to receive information on specific health topics from non-healthcare (food & beauty) services among respondents who had never previously received such information from such source. (Cell denominators vary, total sample N=403)

**Information Type**	**Food establishment**	**Beauty establishment**
Information on healthy lifestyle behaviours	95/368 (25.8%)	85/370 (23.0%)
Information on health screening	98/378 (25.9%)	85/380 (22.4%)
Information on appropriate use of antibiotics	87/380 (22.9%)	83/385 (21.6%)
Information on vaccination	94/376 (25.0%)	87/381 (22.8%)
Information on management of chronic diseases	99/377 (26.3%)	91/384 (23.7%)

Footnote: Due to survey branching logic, respondents who indicated ever receiving information on a given topic from a given source were not asked the willingness follow-up questions and coded as missing. Thus the denominator in each cell equals 403 minus the number who ever-received that topic x source.There was no true item non-response. Values shown as n/N(%).

SURVEY QUESTIONNAIRE

Section 1 – Trust in Health Information Sources

1. On the occasions when you needed advice for health-related matters like medication, diseases, health and general well-being, how much would you **trust** the following sources for information on health?

	Never	Somewhat	Moderately	A lot	Completely
a. Healthcare professionals (e.g. GP, polyclinic doctor, specialist doctor, nurse, pharmacist)	☐ 1	☐ 2	☐ 3	☐ 4	☐ 5
b. Staff of community care centres (e.g. Active Ageing Centre, Dementia Care Centre, Senior Care Centre, Family Service Centre)	☐ 1	☐ 2	☒ 3	☐ 4	☒ 5
c. Non-healthcare professional social contacts (e.g. family member, friend, neighbour)	☐ 1	☐ 2	☐ 3	☐ 4	☐ 5
d. Owners/Staff of food and beverage establishments (e.g. restaurants, cafes, ice cream shops, any other independent eateries)	☒ 1	☐ 2	☐ 3	☐ 4	☐ 5
e. Owners/Staff of beauty establishments (e.g. hair salons/ beauty salons, barber shops)	☐ 1	☐ 2	☐ 3	☐ 4	☐ 5
f. Social influencers/Bloggers (e.g. Mr Brown Annette Lee, Naomi Neo, Benjamin Kheng)	☐ 1	☐ 2	☐ 3	☐ 4	☐ 5

2. On the occasions when you needed advice for health-related matters like medication, diseases, health and general well-being, which of the following sources have you ever **used** to obtain information on health?

	Never	Rarely	Occasionally	Often	Always
a. Healthcare professionals (e.g. GP, polyclinic doctor, specialist doctor, nurse, pharmacist)	☐ 1	☐ 2	☐ 3	☐ 4	☐ 5
b. Community Care centres (e.g. Active Ageing centre, Family Service centre, Dementia Care centre)	☐ 1	☐ 2	☐ 3	☐ 4	☐ 5
c. Non-healthcare professional social contacts (e.g. family member, friend, neighbour)	☐ 1	☐ 2	☐ 3	☐ 4	☐ 5
d. Owners/Staff of food and beverage establishments (e.g. restaurants, cafes, ice cream shops, any other independent eateries)	☐ 1	☐ 2	☐ 3	☐ 4	☐ 5
e. Owners/Staff of beauty establishments (e.g. hair salons/ beauty salons, barber shops)	☐ 1	☐ 2	☐ 3	☐ 4	☐ 5
f. Social influencers/Bloggers (e.g. Mr Brown, Annette Lee, Naomi Neo, Benjamin Kheng)	☐ 1	☐ 2	☐ 3	☐ 4	☐ 5

3. Which of the following type(s) of health information have you **ever received** from the following sources?

	**Information on healthy lifestyle behaviours (e.g. eating a balanced diet, engaging in regular physical activity)**	**Information on health screening (e.g. for diabetes, high blood pressure, high blood cholesterol, cancer)**	**Information on appropriate use of antibiotics (e.g. completing the course of antibiotics as prescribed, not sharing prescribed antibiotics with others)**	**Information on vaccination (e.g. yearly flu vaccination, routine childhood vaccination)**	**Information on management of chronic diseases (e.g. diabetes, high blood pressure, high blood cholesterol, asthma)**
Healthcare professionals (e.g. GP, polyclinic doctor, specialist doctor, nurse, pharmacist)	☐ Yes<br>☐ No, but I am willing to receive this information in the future<br>☐ No. I am also not willing to receive this information in the future	☐ Yes<br>☐ No, but I am willing to receive this information in the future<br>☐ No. I am also not willing to receive this information in the future	☐ Yes<br>☐ No, but I am willing to receive this information in the future<br>☐ No. I am also not willing to receive this information in the future	☐ Yes<br>☐ No, but I am willing to receive this information in the future<br>☐ No. I am also not willing to receive this information in the future	☐ Yes<br>☐ No, but I am willing to receive this information in the future<br>☐ No. I am also not willing to receive this information in the future
Staff of community care centres (e.g. Active Ageing Centre, Dementia Care Centre, Senior Care Centre, Family Service Centre)	☐ Yes<br>☐ No, but I am willing to receive this information in the future<br>☐ No. I am also not willing to receive this information in the future	☐ Yes<br>☐ No, but I am willing to receive this information in the future<br>☐ No. I am also not willing to receive this information in the future	☐ Yes<br>☐ No, but I am willing to receive this information in the future<br>☐ No. I am also not willing to receive this information in the future	☐ Yes<br>☐ No, but I am willing to receive this information in the future<br>☐ No. I am also not willing to receive this information in the future	☐ Yes<br>☐ No, but I am willing to receive this information in the future<br>☐ No. I am also not willing to receive this information in the future
Non-healthcare professional social contacts (e.g. family member, friend, neighbour)	☐ Yes<br>☐ No, but I am willing to receive this information in the future<br>☐ No. I am also not willing to receive this information in the future	☐ Yes<br>☐ No, but I am willing to receive this information in the future<br>☐ No. I am also not willing to receive this information in the future	☐ Yes<br>☐ No, but I am willing to receive this information in the future<br>☐ No. I am also not willing to receive this information in the future	☐ Yes<br>☐ No, but I am willing to receive this information in the future<br>☐ No. I am also not willing to receive this information in the future	☐ Yes<br>☐ No, but I am willing to receive this information in the future<br>☐ No. I am also not willing to receive this information in the future

Owners/Staff of food and beverage establishments (e.g. restaurants, cafes, ice cream shops, any other independent eateries)	☒ Yes<br>☐ No, but I am willing to receive this information in the future<br>☐ No. I am also not willing to receive this information in the future	☐ Yes<br>☐ No, but I am willing to receive this information in the future<br>☐ No. I am also not willing to receive this information in the future	☐ Yes<br>☐ No, but I am willing to receive this information in the future<br>☐ No. I am also not willing to receive this information in the future	☐ Yes<br>☐ No, but I am willing to receive this information in the future<br>☐ No. I am also not willing to receive this information in the future	☐ Yes<br>☐ No, but I am willing to receive this information in the future<br>☐ No. I am also not willing to receive this information in the future
Owners/Staff of beauty establishments (e.g. hair salons/ beauty salons, barber shops)	☐ Yes<br>☐ No, but I am willing to receive this information in the future<br>☐ No. I am also not willing to receive this information in the future	☐ Yes<br>☐ No, but I am willing to receive this information in the future<br>☐ No. I am also not willing to receive this information in the future	☐ Yes<br>☐ No, but I am willing to receive this information in the future<br>☐ No. I am also not willing to receive this information in the future	☐ Yes<br>☐ No, but I am willing to receive this information in the future<br>☐ No. I am also not willing to receive this information in the future	☐ Yes<br>☐ No, but I am willing to receive this information in the future<br>☐ No. I am also not willing to receive this information in the future
Social influencers/Bloggers (e.g. Mr Brown, Annette Lee, Naomi Neo, Benjamin Kheng)	☐ Yes<br>☐ No, but I am willing to receive this information in the future<br>☐ No. I am also not willing to receive this information in the future	☐ Yes<br>☐ No, but I am willing to receive this information in the future<br>☐ No. I am also not willing to receive this information in the future	☐ Yes<br>☐ No, but I am willing to receive this information in the future<br>☐ No. I am also not willing to receive this information in the future	☐ Yes<br>☐ No, but I am willing to receive this information in the future<br>☐ No. I am also not willing to receive this information in the future	☐ Yes<br>☐ No, but I am willing to receive this information in the future<br>☐ No. I am also not willing to receive this information in the future

Section 2 – Health information seeking behaviour

4. On the scale shown, how much do you agree with the following statements?

	Strongly Disagree	Disagree	Neither agree nor disagree	Agree	Strongly Agree
a) I make a point to read and watch stories about health	☐ 1	☐ 2	☐ 3	☐ 4	☐ 5
b) I really enjoy learning about health issues	☐ 1	☐ 2	☐ 3	☐ 4	☐ 5
c) It is critical to be informed about health issues and stay healthy	☐ 1	☐ 2	☐ 3	☐ 4	☐ 5
d) The amount of health information available today make it easier for me to take care of my health	☐ 1	☐ 2	☐ 3	☐ 4	☐ 5
e) When I take medicine, I try to get as much information as possible about its benefits and side-effects	☐ 1	☐ 2	☐ 3	☐ 4	☐ 5
f) I need to know about health issues so I can keep myself and my family healthy	☐ 1	☐ 2	☐ 3	☐ 4	☐ 5
g) Before making a decision about my health, I find out everything I can about this issue	☐ 1	☐ 2	☐ 3	☐ 4	☐ 5
h) It is important for me to be informed about health issues	☐ 1	☐ 2	☐ 3	☐ 4	☐ 5

5. On a scale from very easy to very difficult, how easy would you say it is ...

	Very Easy	Easy	Difficult	Very Difficult
a) To find information about symptoms of illnesses that concern you	☐ 4	☐ 3	☐ 2	☐ 1
b) To find out where to get professional help when you are ill	☐ 4	☐ 3	☐ 2	☐ 1
c) To understand what your doctor says to you	☐ 4	☐ 3	☐ 2	☐ 1
d) To understand your doctor's or pharmacist's instruction on how to take a prescribed medicine	☐ 4	☐ 3	☐ 2	☐ 1
e) To judge if you may need to get a second opinion from another doctor	☐ 4	☐ 3	☐ 2	☐ 1
f) To use information the doctor gives you to make decisions about your illness	☐ 4	☐ 3	☐ 2	☐ 1
g) To follow instructions from your doctor or pharmacist	☐ 4	☐ 3	☐ 2	☐ 1

h)	To find information on how to manage mental health problems like stress or depression	☐ 4	☐ 3	☐ 2	☐ 1
i)	To understand health warnings about behaviour such as smoking, low physical activity and drinking too much	☐ 4	☐ 3	☐ 2	☐ 1
j)	To understand why you need health screenings or examinations	☐ 4	☐ 3	☐ 2	☐ 1
k)	To judge if the information on health risks in the media is reliable	☐ 4	☐ 3	☐ 2	☐ 1
l)	To decide how you can protect yourself from illness using information from the media	☐ 4	☐ 3	☐ 2	☐ 1
m)	To find out about activities that are good for your mental health and well-being	☐ 4	☐ 3	☐ 2	☐ 1
n)	To understand advice concerning your health from family members or friends	☐ 4	☐ 3	☐ 2	☐ 1
o)	To understand information in the media on how to improve your health	☐ 4	☐ 3	☐ 2	☐ 1
p)	To judge which everyday habits affect your health	☐ 4	☐ 3	☐ 2	☐ 1

Section 3 – Socio-demographics

6. What is your residency status?
   - ☐ Singapore citizen
   - ☐ Permanent resident
   - ☐ Foreigner

7. What is your birth year? ____________________________

8. What is your gender?
   - ☐ Female
   - ☐ Male

9. What is your ethnicity?
   - ☐ Chinese
   - ☐ Malay
   - ☐ Indian
   - ☐ Others

10. What is your current marital status?
    - ☐ Never married
    - ☐ Married
    - ☐ Separated
    - ☐ Divorced
    - ☐ Widowed

11. Which of these best describes your household composition?

    - ☐ Single adult only
    - ☐ Single adult and at least 1 child under 21 years

☐ Married and adults only
☐ Married and at least 1 child under 21 years
☐ Multiple adults aged ≥ 21 only
☐ Multiple adults aged ≥ 21 and at least 1 child under 21 years

12. What is your highest educational qualification?
☒ Below secondary
☐ Secondary
☐ Post-secondary (non-tertiary)
☐ Diploma and Professional qualification
☐ University
☐ Post-graduate degree

13. What is your current work status?
☐ Full-time work
☐ Part-time work
☐ Looking for work
☐ Retired
☐ Homemaker
☐ Unemployed (able to work)
☐ Unemployed (unable to work due to medical conditions)
☐ Student
☐ Others (please specify) ________________________

14. Which best describes your current place of residence?
☐ HDB 1- & 2-room flats
☐ HDB 3-room flats
☐ HDB 4-room flats
☐ HDB 5-room & Executive flats
☐ Others (please specify) ________________________

15. When did you start staying in your current residence? ______________ (to state year)

16. How would you rate your current level of health?
☐ Very good
☐ Good
☐ Fair
☐ Poor
☐ Very poor
